# Supplementary material for: Structural regulation of halide superionic conductors for all-solid-state lithium batteries
Source: Nat Commun. 2024 Jan 2;15:53. doi: 10.1038/s41467-023-43886-9 (PMC10761688; doi:10.1038/s41467-023-43886-9)
Supplement: Supplementary file 1 — Supporting information [file 41467_2023_43886_MOESM1_ESM.pdf]

(Supplementary Information)

Structural Regulation of Halide Superionic Conductors for All-Solid-State Lithium Batteries

Xiaona Li[1,2], Jung Tae Kim[2], Jing Luo[2], Changtai Zhao[3], Yang Xu[3,4], Tao Mei[4], Ruying Li[2], Jianwen Liang[2,3]*, and Xueliang Sun[2,]*

[1]Eastern Institute for Advanced Study, Eastern Institute of Technology, Ningbo, Zhejiang 315200, P. R. China.

[2]Department of Mechanical and Materials Engineering, University of Western Ontario, 1151 Richmond St, London, Ontario, N6A 3K7, Canada.

[3]Solid State Batteries Research Center, GRINM (Guangdong) Institute for Advanced Materials and Technology, Foshan, Guangdong, 528051, P. R. China.

[4]School of Materials Science and Engineering, Hubei University, Wuhan 430062, P. R. China

*Corresponding email: liangjianwen@grinm.com, xsun9@uwo.ca

The dividing line to distinguish *hcp* and *ccp* Li-M-X halides based on the cation and anion radii

The dividing lines are obtained based on the representative hcp-type and ccp-type Li-M-X halides, taking into consideration the composition of metal cations, and the radius of cations and anions (Table S1). The dividing line is fitted as y = 0.647x-0.079. While due to the limited number of examples, there may be an error in the present dividing line.

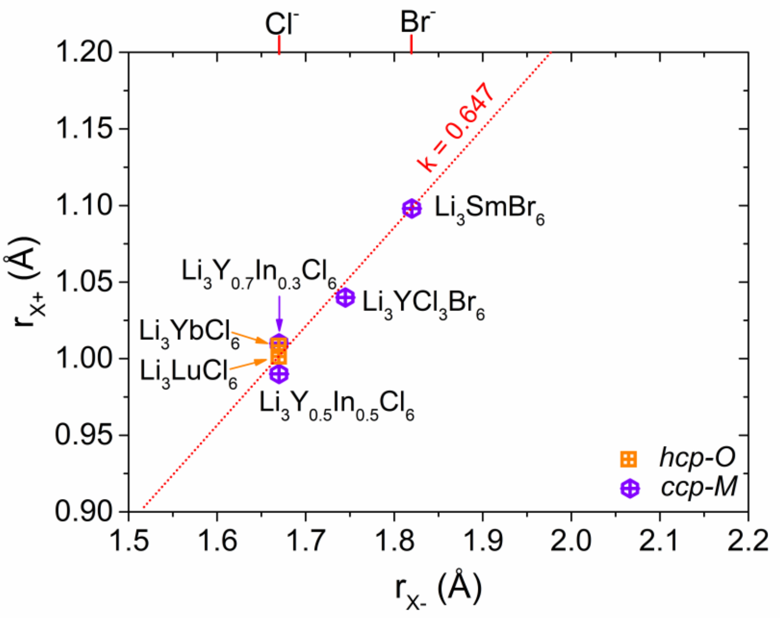

Supplementary Fig. 1 The dividing line to distinguish *hcp* and *ccp* Li_aMX_b based on the cation and anion radius.

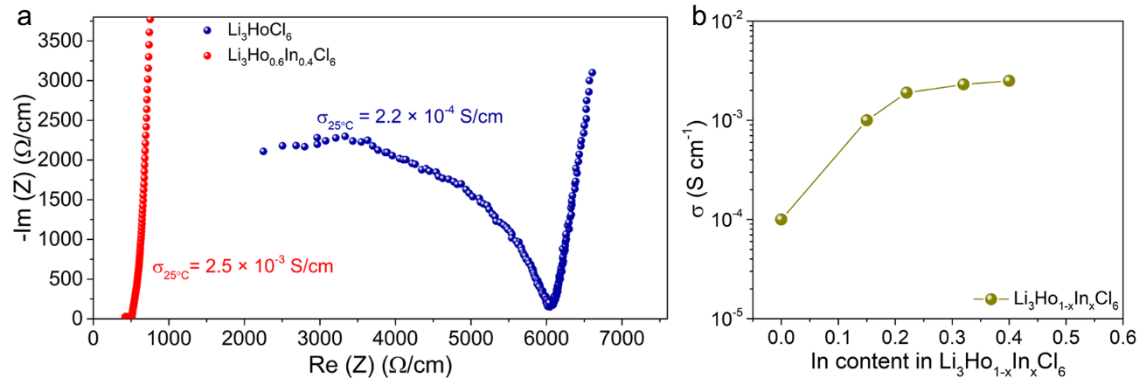

Supplementary Fig. 2 (a) The EIS spectra of typical $Li_3Ho_{1-x}In_xCl_6$ halides at 25 ℃. (b) The ionic conductivity evolution of the $Li_3Ho_{1-x}In_xCl_6$ halides at 25 ℃.

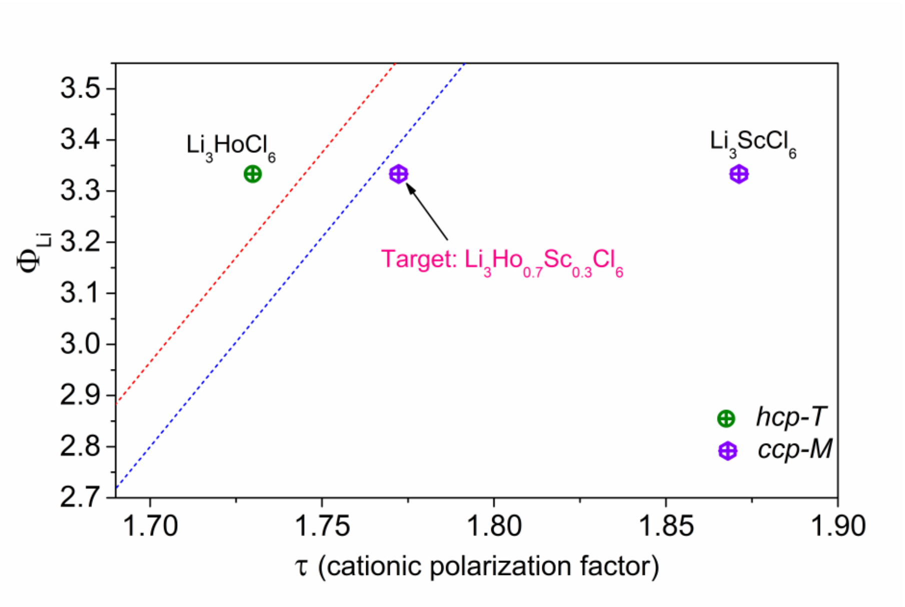

Supplementary Fig. 3 Analysis of the cationic polarization factor of $Li_3Ho_{1-x}Sc_xCl_6$ halides.

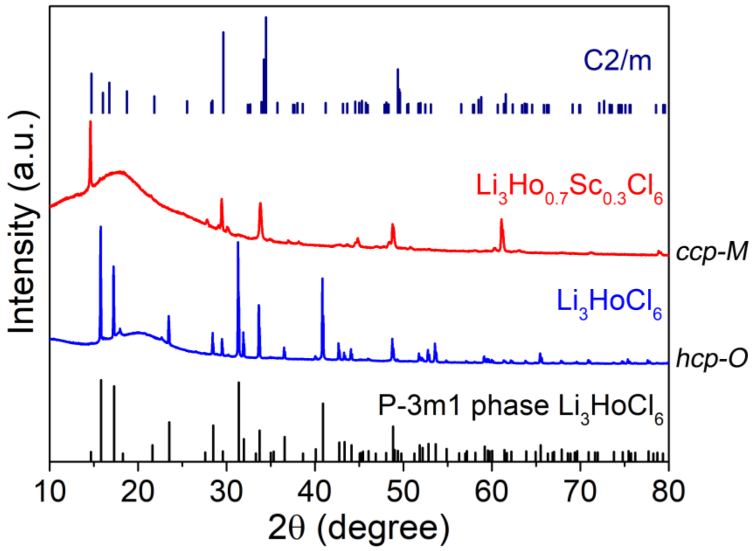

Supplementary Fig. 4 XRD patterns of the targeted $Li_3Ho_{1-x}Sc_xCl_6$ samples and the standard references.

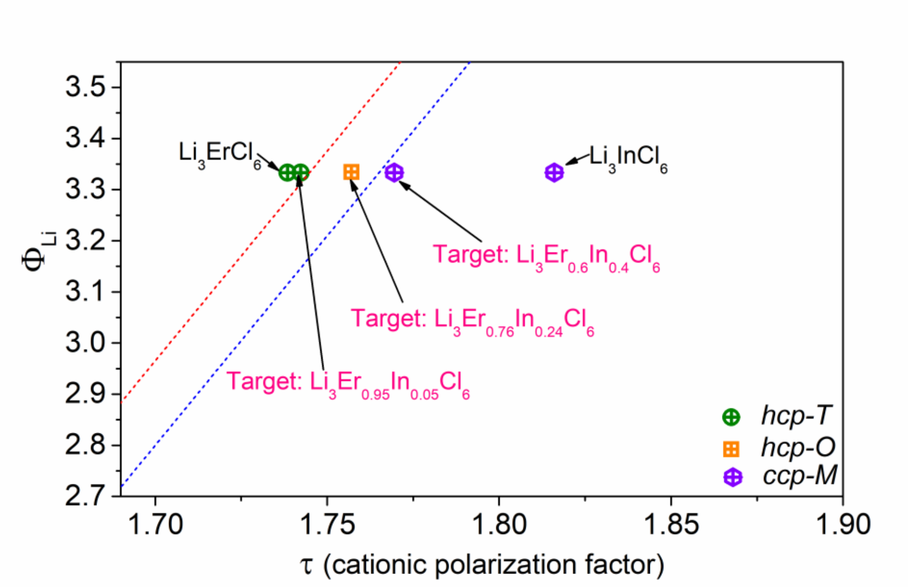

Supplementary Fig. 5 Analysis of the cationic polarization factor of $Li_3Er_{1-x}In_xCl_6$ halides.

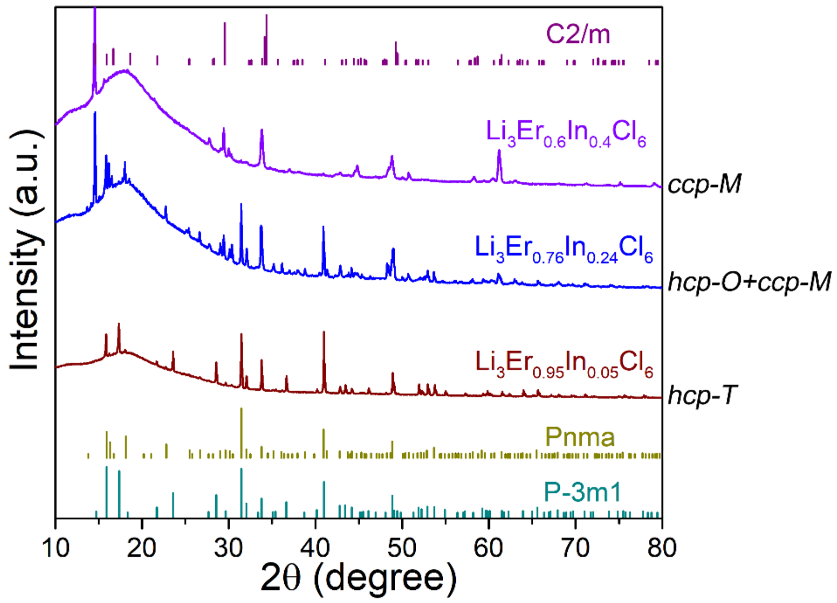

Supplementary Fig. 6 XRD patterns of the targeted $Li_3Er_{1-x}In_xCl_6$ halides and the standard references.

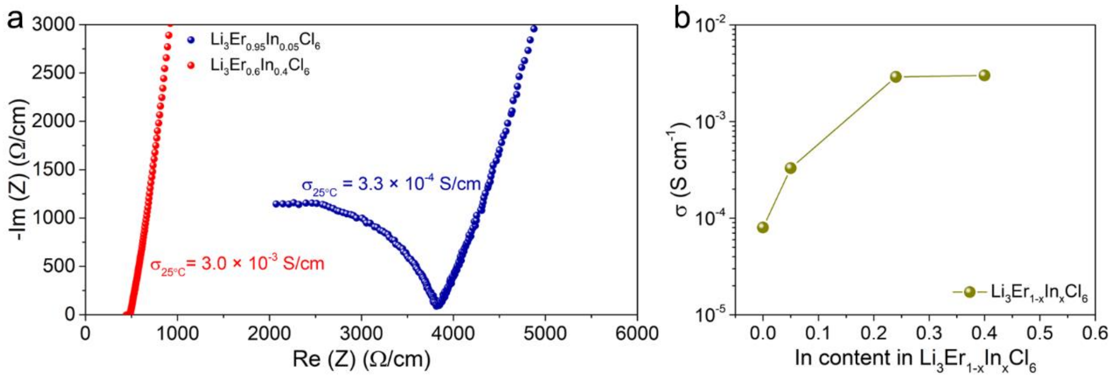

Supplementary Fig. 7 (a) The EIS spectra of typical $Li_3Er_{1-x}In_xCl_6$ halides at 25 °C. (b) The ionic conductivity evolution of the $Li_3Er_{1-x}In_xCl_6$ halides at 25 °C.

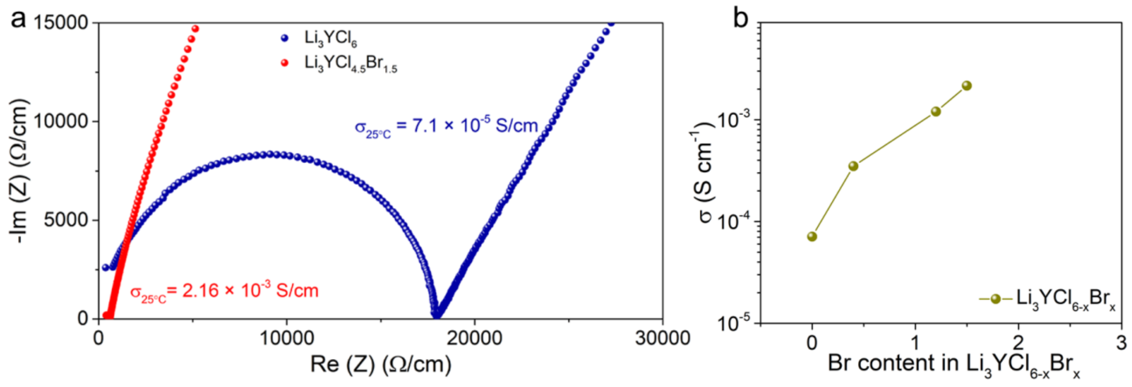

Supplementary Fig. 8 (a) The EIS spectra of typical $Li_3YCl_{6-x}Br_x$ halides at 25 °C. (b) The ionic conductivity evolution of the $Li_3YCl_{6-x}Br_x$ halides at 25 °C.

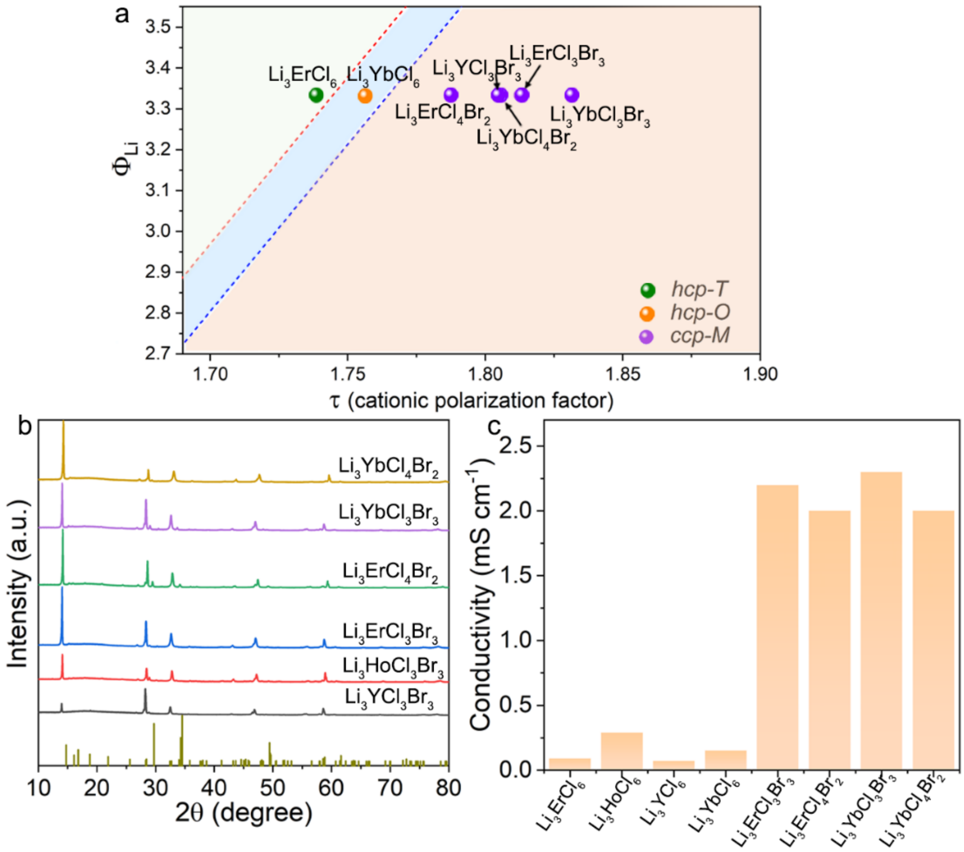

Supplementary Fig. 9 Analysis of the cationic polarization factor of $Li_3ErCl_{6-x}Br_x$ and $Li_3YbCl_{6-x}Br_x$ halides. (b) XRD pattern of $Li_3HoCl_3Br_3$, $Li_3YCl_3Br_3$, $Li_3ErCl_{1-x}Br_x$, and $Li_3YbCl_{6-x}Br_x$ halides. (c) The ionic conductivity evolution of the $Li_3ErCl_{1-x}Br_x$ and $Li_3YbCl_{6-x}Br_x$ halides at 25 °C.

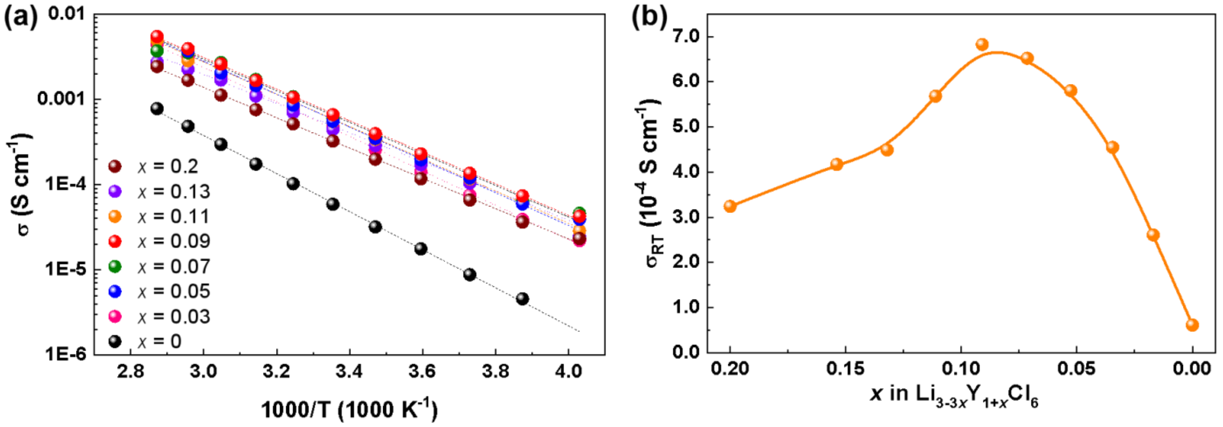

Supplementary Fig. 10 (a) The Arrhenius plots of typical $Li_{3-3x}Y_{1-x}Cl_6$ halides. (b) The ionic conductivity evolution of the $Li_{3-3x}Y_{1-x}Cl_6$ halides at 25 °C.

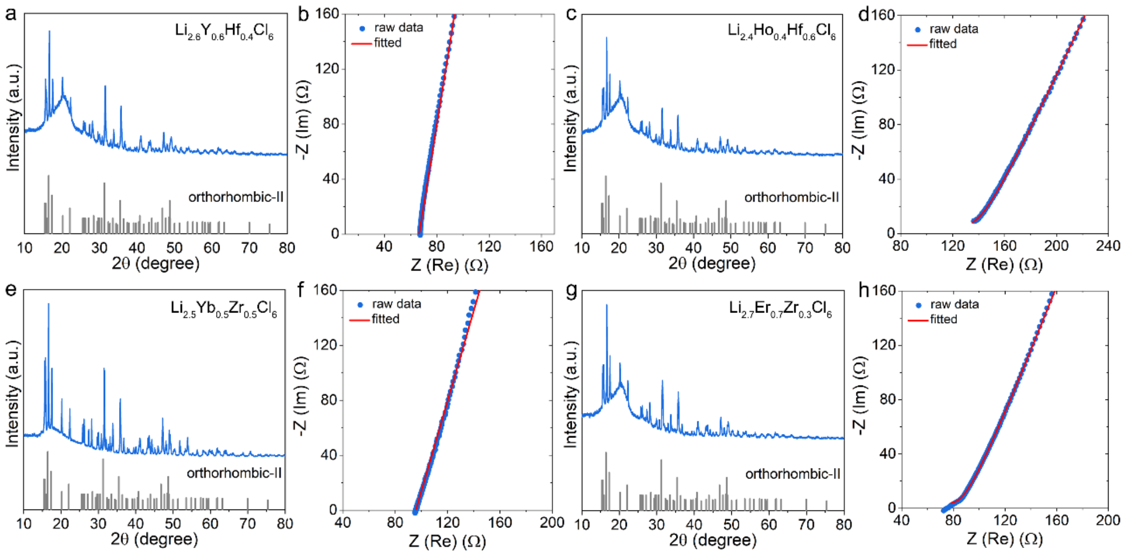

Supplementary Fig. 11. (a) XRD pattern and (b) EIS spectra of $Li_{2.6}Y_{0.6}Hf_{0.4}Cl_6$ (thickness of 0.7 mm), (a) XRD pattern and (b) EIS spectra of $Li_{2.4}Ho_{0.4}Hf_{0.6}Cl_6$ (thickness of 0.8 mm), (a) XRD pattern and (b) EIS spectra of $Li_{2.5}Yb_{0.5}Zr_{0.5}Cl_6$ (thickness of 1.2 mm), (a) XRD pattern and (b) EIS spectra of $Li_{2.7}Er_{0.7}Zr_{0.3}Cl_6$ (thickness of 0.8 mm).

Supplementary Table 1. The summary of the cation, halogen anion crystal radius, and their ratio of the typical Li-M-X halides.

Halides	cation Radius (Å)	Halogen anion Radius (Å)	radius ratio of X^-	Ref.
Li_3ScF_6	0.885	1.19	0.7437	1
$LiYF_4$	1.04	1.19	0.8740	2
$LiEuF_4$	1.087	1.19	0.9135	2
$LiGdF_4$	1.078	1.19	0.9059	2
$LiTbF_4$	1.063	1.19	0.8933	2
$LiDyF_4$	1.052	1.19	0.8840	2
$LiHoF_4$	1.041	1.19	0.8748	3
$LiErF_4$	1.03	1.19	0.8656	3
$LiTmF_4$	1.02	1.19	0.8571	2
$LiYbF_4$	1.008	1.19	0.8471	2
$LiLuF_4$	1.001	1.19	0.8412	2
Li_3InCl_6	0.94	1.67	0.5629	4
Li_3ScCl_6	0.885	1.67	0.5299	5
Li_3YCl_6	1.04	1.67	0.6228	6
$LiGdCl_4$	1.078	1.67	0.6455	7
Li_3TbCl_6	1.063	1.67	0.6365	6
Li_3DyCl_6	1.052	1.67	0.6299	6
Li_3HoCl_6	1.041	1.67	0.6234	6
Li_3ErCl_6	1.03	1.67	0.6168	6

Li_3TmCl_6	1.02	1.67	0.6108	[6]
Li_3YbCl_6	1.008	1.67	0.6036	[6]
Li_3LuCl_6	1.001	1.67	0.5994	[6]
Li_2ZrCl_6	0.86	1.67	0.5150	[8]
Li_3InBr_6	0.94	1.82	0.5165	[9]
Li_3ScBr_6	0.885	1.82	0.4863	[10]
Li_3YBr_6	1.04	1.82	0.5714	[11]
Li_3SmBr_6	1.098	1.82	0.6033	[11]
Li_3EuBr_6	1.087	1.82	0.5973	[11]
Li_3GdBr_6	1.078	1.82	0.5923	[11]
Li_3TbBr_6	1.063	1.82	0.5841	[11]
Li_3DyBr_6	1.052	1.82	0.5780	[11]
Li_3HoBr_6	1.041	1.82	0.5720	[11,12]
Li_3ErBr_6	1.03	1.82	0.5659	[11]
Li_3TmBr_6	1.02	1.82	0.5604	[11]
Li_3YbBr_6	1.008	1.82	0.5539	[11]
Li_3LuBr_6	1.001	1.82	0.5500	[11]
Li_3InI_6	0.94	2.06	0.4563	
$LiScI_3$	0.885	2.06	0.4296	[13]
Li_3ErI_6	1.03	2.06	0.5000	[14]
Li_2ZrI_6	0.86	2.06	0.4175	[15]

Supplementary Note 1 The structures of Li_aMX_b halide discussed in this work are focused on entropy-dominated phases, possible metastable structures are not included (such as *hcp-T* type Li_2ZrCl_6 synthesized by mechanical milling method[8]). Moreover, it's noted that the M^{3+} in the $LiMF_4$ here is still six-coordinated, so the radius calculated here is still the same as in the Li_3MX_6 structure.

Supplementary Table 2. The calculation of the $k = 0.647$ in **Fig. 1e** is based on the cation and anion radii ratio of several halides around the critical line, including Li_3SmBr_6, $Li_3Y_{0.5}In_{0.5}Cl_6$, $Li_3Y_{0.7}In_{0.3}Cl_6$, $Li_3YBr_3Cl_3$, Li_3YbCl_6, and Li_3LuCl_6. Note: For Li_3MX_6 halides with multiple metal cations or anions, the radius is average radius based on the content of each component.

Halides	Cation radius (Å)	Anion radius (Å)	Structure	Ref.
Li_3SmBr_6	1.098	1.82	*ccp-M*	[11]
$Li_3Y_{0.5}In_{0.5}Cl_6$	0.99	1.67	*ccp-M*	[16]
$Li_3Y_{0.7}In_{0.3}Cl_6$	1.01	1.67	*ccp-M*	[16]
$Li_3YBr_3Cl_3$	1.04	1.745	*ccp-M*	[17]
Li_3YbCl_6	1.008	1.67	*hcp-O*	[6]
Li_3LuCl_6	1.001	1.67	*hcp-O*	[6]

Supplementary Table 3. Charge, crystal ionic radius (Å), and ionic potential of the elements in Li_aMX_b halide SSEs. Ions are 6-coordinate unless indicated.

Ion	Ionic Charge	Crystal ionic radius (Å)	Ionic potential ($Å^{-1}$)
Li^+	1	0.9	1.1111
In^{3+}	3	0.94	3.1915
Sc^{3+}	3	0.885	3.3898
Y^{3+}	3	1.04	2.8846
La^{3+}	3	1.172	2.5597
Ce^{3+}	3	1.15	2.6087
Pr^{3+}	3	1.13	2.6549
Nd^{3+}	3	1.123	2.6714
Pm^{3+}	3	1.11	2.7027
Sm^{3+}	3	1.098	2.7322
Eu^{3+}	3	1.087	2.7600
Gd^{3+}	3	1.078	2.7829
Tb^{3+}	3	1.063	2.8222
Dy^{3+}	3	1.052	2.8517
Ho^{3+}	3	1.041	2.8818
Er^{3+}	3	1.03	2.9126
Tm^{3+}	3	1.02	2.9412
Yb^{3+}	3	1.008	2.9762
Lu^{3+}	3	1.001	2.9970
Zr^{4+}	4	0.86	4.6512
Hf^{4+}	4	0.85	4.7059
F^-	1	1.19	0.8403
Cl^-	1	1.67	0.5988
Br^-	1	1.82	0.5495
I^-	1	2.06	0.4854

Supplementary Table 4. Calculated molar content proportionally ionic potential of different ions and corresponding cationic polarization factors for Li_aMX_b halides. The reported conductivities of Li_aMX_b halides are also presented.

Halides	Structure	$\tau = (\Phi Li + \Sigma\Phi M)/\Sigma\phi X$	$\phi Li = n_{Li}\cdot I_{Li}$ ($Å^{-1}$)	$\phi M = n_M\cdot I_M$ ($Å^{-1}$)	$\phi X = n_X\cdot I_X$ ($Å^{-1}$)	Conductivity (S cm^{-1}, 25 °C)	Ref.
Li_3YCl_6	hcp-T	1.7306	3.3333	2.8846	3.5928	7.1×10^{-5}	[18]
Li_3TbCl_6	hcp-T	1.7133	3.3333	2.8222	3.5928	2.2×10^{-4}	[19]
Li_3DyCl_6	hcp-T	1.7215	3.3333	2.8517	3.5928	1.2×10^{-4}	[19]

Li_3HoCl_6	hcp-T	1.7300	3.3333	2.8818	3.5928	2.9×10^{-4}	[6]
Li_3ErCl_6	hcp-T	1.7385	3.3333	2.9126	3.5928	9×10^{-5}	[20]
Li_3TmCl_6	hcp-T	1.7464	3.3333	2.9412	3.5928	1.1×10^{-4}	[6]
$Li_3Er_{0.95}In_{0.05}Cl_6$	hcp-T	1.7423	3.3333	2.9265	3.5928	3.3×10^{-4}	This work
Li_3YbCl_6	hcp-O	1.7562	3.3333	2.9762	3.5928	1.5×10^{-4}	[6,21]
Li_3LuCl_6	hcp-O	1.7619	3.3333	2.9970	3.5928	-	[6]
$Li_{2.727}Dy_{1.091}Cl_6$	hcp-O	1.7092	3.0300	3.1109	3.5928	9×10^{-4}	[19]
$Li_{2.727}Ho_{1.091}Cl_6$	hcp-O	1.7184	3.0300	3.1438	3.5928	1.3×10^{-3}	[19]
$Li_{2.727}Er_{1.091}Cl_6$	hcp-O	1.7277	3.0300	3.1774	3.5928	6.4×10^{-4}	[19]
$Li_{2.727}Y_{1.091}Cl_6$	hcp-O	1.7193	3.0300	3.1471	3.5928	6.9×10^{-4}	[19]
$Li_{2.727}Tm_{1.091}Cl_6$	hcp-O	1.7365	3.0300	2.9412	3.5928	8.9×10^{-4}	[19]
$Li_{2.82}Ho_{1.06}Cl_6$	hcp-O	1.7223	3.1333	3.0547	3.5928	9.4×10^{-4}	This work
$Li_{2.83}Y_{1.057}Cl_6$	hcp-O	1.7238	3.1444	3.049	3.5928	5.7×10^{-4}	This work
$Li_{2.911}Er_{1.03}Cl_6$	hcp-O	1.7353	3.2344	3	3.5928	4.9×10^{-4}	This work
$Li_{2.93}(Ho_{0.8}In_{0.2})_{1.023}Cl_6$	hcp-O	1.7448	3.2555	3.0131	3.5928	1.3×10^{-3}	This work
Li_3HoCl_5Br	hcp-O	1.7539	3.3333	2.8818	3.5435	1.3×10^{-3}	This work
Li_3ErCl_5Br	hcp-O	1.7626	3.3333	2.9126	3.5435	1.14×10^{-3}	This work
$Li_3YCl_{4.8}Br_{1.2}$	hcp-O	1.7597	3.3333	2.8846	3.5336	1.2×10^{-3}	This work
$Li_{2.75}Yb_{0.75}Hf_{0.25}Cl_6$	hcp-O	1.7992	3.0555	3.4086	3.5928	7×10^{-4}	[21]
$Li_{2.8}Yb_{0.8}Zr_{0.2}Cl_6$	hcp-O	1.7876	3.1111	3.3112	3.5928	1.1×10^{-3}	[22]
$Li_{2.7}Yb_{0.7}Zr_{0.3}Cl_6$	hcp-O	1.8033	3.0000	3.4787	3.5928	1.1×10^{-3}	[22]
$Li_{2.5}Yb_{0.5}Zr_{0.5}Cl_6$	hcp-O	1.8346	2.7778	3.8137	3.5928	1.1×10^{-3}	[22]
$Li_{2.5}Y_{0.5}Zr_{0.5}Cl_6$	hcp-O	1.8219	2.7778	3.7679	3.5928	1.4×10^{-3}	[23]
$Li_{2.4}Y_{0.4}Zr_{0.6}Cl_6$	hcp-O	1.8401	2.6666	3.9446	3.5928	9.5×10^{-4}	[23]
$Li_{2.633}Er_{0.633}Zr_{0.367}Cl_6$	hcp-O	1.8026	2.9255	3.5507	3.5928	1.1×10^{-3}	[23]
$Li_{2.4}Er_{0.4}Zr_{0.6}Cl_6$	hcp-O	1.8432	2.6666	3.9558	3.5928	8×10^{-4}	[23]
Li_3YBr_6	ccp-M	1.8861	3.3333	2.8846	3.2967	1.7×10^{-3}	[18]
$Li_3YBr_{5.7}F_{0.3}$	ccp-M	1.8373	3.3333	2.8846	3.3842	1.8×10^{-3}	[24]
$Li_3Y_{0.5}In_{0.5}Cl_6$	ccp-M	1.7734	3.3333	3.0381	3.5928	1.22×10^{-3}	[16]
$Li_3Y_{0.3}In_{0.7}Cl_6$	ccp-M	1.7904	3.3333	3.0994	3.5928	1.17×10^{-3}	[16]
$Li_3Y_{0.1}In_{0.9}Cl_6$	ccp-M	1.8075	3.3333	3.1608	3.5928	1.15×10^{-3}	[16]
$Li_3YBr_3Cl_3$	ccp-M	2.0027	3.3333	2.8846	3.1047	7.2×10^{-3}	[17]

Li_3InCl_6	ccp-M	1.8161	3.3333	3.1915	3.5928	2×10^{-3}	[4]
Li_3ScCl_6	ccp-M	1.8713	3.3333	3.3898	3.5928	3×10^{-3}	[5]
$Li_{2.727}Sc_{1.091}Cl_6$	ccp-M	1.8727	3.0300	3.6983	3.5928	1.98×10^{-3}	[5]
$Li_{2.727}In_{1.091}Cl_6$	ccp-M	1.8125	3.0300	3.4819	3.5928	1×10^{-3}	This work
Li_2ZrCl_6 (heat)	ccp-M	1.9131	2.2222	4.6512	3.5928	6×10^{-6}	[8,25]
$Li_{2.9}In_{0.9}Zr_{0.1}Cl_6$	ccp-M	1.8258	3.2222	3.3375	3.5928	1.54×10^{-3} (20°C)	[26,27]
$Li_{2.85}In_{0.85}Zr_{0.15}Cl_6$	ccp-M	1.8281	3.1666	3.4105	3.5928	1.26×10^{-3} (20°C)	[26,27]
$Li_{2.6}In_{0.6}Zr_{0.4}Cl_6$	ccp-M	1.8549	2.8889	3.7754	3.5928	1.8×10^{-3}	[28]
$Li_{2.5}In_{0.5}Zr_{0.5}Cl_6$	ccp-M	1.8646	2.7778	3.9214	3.5928	1.8×10^{-3}	[28]
$Li_{2.8}In_{0.8}Zr_{0.2}Cl_6$	ccp-M	1.8355	3.1111	3.4834	3.5928	1.4×10^{-3}	[28]
Li_3InBr_6	ccp-M	1.9792	3.3333	3.1915	3.2967	1×10^{-3} (high-temperature phase)	[29]
Li_3ScBr_6	ccp-M	2.0393	3.3333	3.3898	3.2967	-	[10]
Li_3HoBr_6	ccp-M	1.8853	3.3333	2.8818	3.2967	1.25×10^{-3}	[11,12]
Li_3SmBr_6	ccp-M	1.8399	3.3333	2.7322	3.2967	-	[11]
Li_3EuBr_6	ccp-M	1.8483	3.3333	2.7600	3.2967	-	[11]
Li_3GdBr_6	ccp-M	1.8553	3.3333	2.7829	3.2967	-	[11]
Li_3TbBr_6	ccp-M	1.8672	3.3333	2.8222	3.2967	-	[11]
Li_3DyBr_6	ccp-M	1.8761	3.3333	2.8517	3.2967	-	[11]
Li_3ErBr_6	ccp-M	1.8946	3.3333	2.9126	3.2967	-	[11]
Li_3TmBr_6	ccp-M	1.9033	3.3333	2.9412	3.2967	-	[11]
Li_3YbBr_6	ccp-M	1.9139	3.3333	2.9762	3.2967	-	[11]
Li_3LuBr_6	ccp-M	1.9202	3.3333	2.9970	3.2967	-	[11]
$Li_3HoBr_3I_3$	ccp-M		3.3333	2.8818	3.1047	2.7 (20°C)	[30]
Li_3HoI_6	ccp-M	2.1340	3.3333	2.8818	2.9124	1.5×10^{-4} (20°C)	[11,30]
$Li_{2.85}(Ho_{0.5}In_{0.5})_{1.05}Cl_6$	ccp-M	1.7688	3.1666	3.1884	3.5928	3.1×10^{-3}	This work
$Li_3HoCl_3Br_3$	ccp-M	1.8042	3.3333	2.8818	3.4449	4.1×10^{-3a}	This work
$Li_3HoCl_4Br_2$	ccp-M	1.7787	3.3333	2.8818	3.4942	2.3×10^{-3}	This work
$Li_3ErCl_3Br_3$	ccp-M	1.8131	3.3333	2.9126	3.4449	2.2×10^{-3}	This work
$Li_3ErCl_4Br_2$	ccp-M	1.7875	3.3333	2.9126	3.4942	2.0×10^{-3}	This work
$Li_3YbCl_3Br_3$	ccp-M	1.8316	3.3333	2.9762	3.4449	2.3×10^{-3}	This work
$Li_3YbCl_4Br_2$	ccp-M	1.8057	3.3333	2.9762	3.4942	2.0×10^{-3}	This work

$Li_3YCl_{4.5}Br_{1.5}$	ccp-M	1.7670	3.3333	2.8846	3.5189	2.1×10^{-3}	This work
$Li_3Er_{0.76}In_{0.24}Cl_6$	hcp-O+ ccp-M	1.7571	3.3333	2.9795	3.5928	2.9×10^{-3}	This work
$Li_3Er_{0.6}In_{0.4}Cl_6$	ccp-M	1.7696	3.3333	3.0242	3.5928	3×10^{-3}	This work
$Li_3Ho_{0.7}Sc_{0.3}Cl_6$	ccp-M	1.7723	3.3333	3.0342	3.5928	4.7×10^{-3}[a]	This work
$Li_3Ho_{0.78}In_{0.22}Cl_6$	hcp-O+ ccp-M	1.7488	3.3333	2.9499	3.5928	1.9×10^{-3}	This work
$Li_3Ho_{0.68}In_{0.32}Cl_6$	ccp-M	1.7575	3.3333	2.9809	3.5928	2.3×10^{-3}	This work
$Li_3Ho_{0.6}In_{0.4}Cl_6$	ccp-M	1.7644	3.3333	3.0057	3.5928	2.5×10^{-3}	This work

a. Data recorded for hot-pressed solid electrolyte pellet.

Supplementary Table 5. Examples of *hcp-T*, *hcp-O*, and *ccp-M* type halides in $Li_3Ho_{1-x}InCl_6$ system based on the proposed cationic polarization factor.

Halides	$\tau = (\Phi Li + \Sigma\Phi M)/\Sigma\phi X$	$\phi Li = n_{Li}\cdot I_{Li}$ ($Å^{-1}$)	$\phi M = n_M\cdot I_M$ ($Å^{-1}$)	$\phi X = n_X\cdot I_X$ ($Å^{-1}$)	Structure	Ref.
Li_3HoCl_6	1.7299	3.3333	2.8818	3.5928	hcp-T	[6]
$Li_3Ho_{0.85}In_{0.15}Cl_6$	1.7428	3.3333	2.9283	3.5928	hcp-T+ hcp-O	This work
$Li_3Ho_{0.78}In_{0.22}Cl_6$	1.7488	3.3333	2.9499	3.5928	hcp-O+ ccp-M	This work
$Li_3Ho_{0.6}In_{0.4}Cl_6$	1.7644	3.3333	3.0057	3.5928	ccp-M	This work
Li_3InCl_6	1.8161	3.3333	3.1915	3.5928	ccp-M	[4]

Supplementary Table 6. Rietveld refinement of XRD pattern of *ccp-M* typed $Li_3Ho_{0.6}In_{0.4}Cl_6$.

Compound	$Li_6Ho_{1.203}In_{0.797}Cl_{12}$
Space Group	C2/m
a, Å	6.523(70)
b, Å	11.217(50)
c, Å	6.437(90)
α=γ, °	90
β	109.793
V, $Å^3$	443.290
2θ interval, °	10 - 90

Rwp,%	4.88
Rp, %	3.41
Radiation	Cu kα<br>λ1 = 1.5406 Å, λ2 = 1.5444 Å

Supplementary Table 7. The atom sites of *ccp-M* typed $Li_3Ho_{0.6}In_{0.4}Cl_6$.

Atom	x	y	z	Occ.	U	site
Ho	0.000000	0.315480	0.000000	0.4423	0.038	4g
In	0.000000	0.000000	0.000000	0.1154	0.284	2a
Cl	0.245000	0.000000	-0.233800	1.0000	0.074	4i
Li	0.500000	0.000000	0.500000	1.0000	0.020	2d
Li	0.000000	0.168300	0.500000	1.0000	0.089	4h
Cl	0.242100	0.162200	0.238800	1.0000	0.012	8j

Supplementary Table 8. Examples of *hcp-T* and *ccp-M* type halides in $Li_3Ho_{1-x}Sc_xCl_6$ system based on the proposed cationic polarization factor.

Halides	$\tau = (\Phi Li + \Sigma\Phi M)/\Sigma\phi X$	$\phi Li = n_{Li}\cdot I_{Li}$ ($Å^{-1}$)	$\phi M = n_M\cdot I_M$ ($Å^{-1}$)	$\phi X = n_X\cdot I_X$ ($Å^{-1}$)	Structure	Ref.
Li_3HoCl_6	1.7299	3.3333	2.8818	3.5928	hcp-T	[6]
$Li_3Ho_{0.7}Sc_{0.3}Cl_6$	1.7722	3.3333	3.0342	3.5928	ccp-M	This work
Li_3ScCl_6	1.8713	3.3333	3.3898	3.5928	ccp-M	[5]

Supplementary Table 9. Examples of *hcp-T*, *hcp-O*, and *ccp-M* type halides in $Li_3Er_{1-x}In_xCl_6$ system based on the proposed cationic polarization factor.

Halides	$\tau = (\Phi Li + \Sigma\Phi M)/\Sigma\phi X$	$\phi Li = n_{Li}\cdot I_{Li}$ ($Å^{-1}$)	$\phi M = n_M\cdot I_M$ ($Å^{-1}$)	$\phi X = n_X\cdot I_X$ ($Å^{-1}$)	Structure	Ref.
Li_3ErCl_6	1.7385	3.3333	2.9126	3.5928	hcp-T	[20]
$Li_3Er_{0.95}In_{0.05}Cl_6$	1.7423	3.3333	2.9265	3.5928	hcp-T	This work
$Li_3Er_{0.76}In_{0.24}Cl_6$	1.7571	3.3333	2.9795	3.5928	hcp-O+ ccp-M	This work
$Li_3Er_{0.6}In_{0.4}Cl_6$	1.7695	3.3333	3.0242	3.5928	ccp-M	This work
Li_3InCl_6	1.8161	3.3333	3.1915	3.5928	ccp-M	[4]

Supplementary Table 10. Examples of *hcp-T*, *hcp-O*, and *ccp-M* type halides in $Li_3YCl_{6-x}Br_x$ system based on the proposed cationic polarization factor.

Halides	τ = (ΦLi + ΣΦM)/ΣϕX	ϕLi = $n_{Li}·I_{Li}$ (Å^{-1})	ϕM = $n_M·I_M$ (Å^{-1})	ϕX = $n_X·I_X$ (Å^{-1})	Structure	Ref.
Li_3YCl_6	1.7307	3.3333	2.8846	3.5928	hcp-T	[18]
$Li_3YCl_{5.6}Br_{0.4}$	1.7402	3.3333	2.8846	3.5731	hcp-T	This work
$Li_3YCl_{4.8}Br_{1.2}$	1.7597	3.3333	2.8846	3.5336	hcp-O	This work
$Li_3YCl_{4.5}Br_{1.5}$	1.7670	3.3333	2.8846	3.5189	ccp-M	This work
$Li_3YCl_3Br_3$	1.8050	3.3333	2.8846	3.4449	ccp-M	[17]
Li_3YBr_6	1.8861	3.3333	2.8846	3.2967	ccp-M	[18]

Supplementary Table 11. Rietveld refinement of XRD pattern of *hcp-O* typed $Li_3YCl_{4.8}Br_{1.2}$.

Compound	$Li_3YCl_{4.82}Br_{1.18}$
Space Group	Pnma
a, Å	12.971(85)
b, Å	11.182(67)
c, Å	6.044(18)
α=β=γ, °	90
V, Å^3	876.768
2θ interval, °	10 - 90
Rwp,%	3.1
Rp, %	3.41
Radiation	Cu kα<br>λ1 = 1.5406 Å, λ2 = 1.5444 Å

Supplementary Table 12. The atom sites of *hcp-O* typed $Li_3YCl_{4.8}Br_{1.2}$.

Atom	x	y	z	Occ.	U	site
**Li**	0.11680	0.07660	0.00570	0.750	0.007	8d
**Li**	0.13680	0.09140	0.50610	0.750	0.007	8d
**Cl**	0.21351	0.59871	0.27398	0.701	0.054	8d
**Cl**	0.45858	0.07327	0.25118	0.710	0.054	8d
**Cl**	0.04320	0.25000	0.72870	1.000	0.010	4c
**Cl**	0.20480	0.25000	0.23960	1.000	0.010	4c
**Y**	0.37560	0.25000	0.01070	1.000	0.023	4c
**Br**	0.21351	0.59871	0.27398	0.299	0.054	8d

Supplementary Table 13. Critical points of $Li_{3-3x}Dy_{1+x}Cl_6$, $Li_{3-3x}Ho_{1+x}Cl_6$, $Li_{3-3x}Y_{1+x}Cl_6$, and $Li_{3-3x}Er_{1+x}Cl_6$ halides from *hcp-T*, to *hcp-O* structure based on the proposed cationic polarization factor.

Halides	$\tau = (\Phi Li + \Sigma\Phi M)/\Sigma\phi X$	$\phi Li = n_{Li}\cdot I_{Li}$ (Å^{-1})	$\phi M = n_M\cdot I_M$ (Å^{-1})	$\phi X = n_X\cdot I_X$ (Å^{-1})	Structure	Ref.
Li_3TbCl_6	1.7133	3.3333	2.8222	3.5928	hcp-T	[6]
Li_3DyCl_6	1.7215	3.3333	2.8517	3.5928	hcp-T	[6]
Li_3HoCl_6	1.7300	3.3333	2.8818	3.5928	hcp-T	[6]
Li_3YCl_6	1.7306	3.3333	2.8846	3.5928	hcp-T	[6]
Li_3ErCl_6	1.7385	3.3333	2.9126	3.5928	hcp-T	[6]
Li_3TmCl_6	1.7464	3.3333	2.9412	3.5928	hcp-T	[6]
Li_3YbCl_6	1.7562	3.3333	2.9762	3.5928	hcp-O	[6]
Li_3LuCl_6	1.7619	3.3333	2.9970	3.5928	hcp-O	[6]
$Li_{2.727}Dy_{1.091}Cl_6$	1.7092	3.0300	3.1109	3.5928	hcp-O	[19]
$Li_{2.727}Ho_{1.091}Cl_6$	1.7184	3.0300	3.1438	3.5928	hcp-O	[19]
$Li_{2.727}Y_{1.091}Cl_6$	1.7193	3.0300	3.1471	3.5928	hcp-O	[19]
$Li_{2.727}Er_{1.091}Cl_6$	1.7277	3.0300	3.1774	3.5928	hcp-O	[19]
$Li_{2.82}Ho_{1.06}Cl_6$	1.7223	3.1333	3.0547	3.5928	hcp-O	This work
$Li_{2.83}Y_{1.057}Cl_6$	1.7238	3.1444	3.0490	3.5928	hcp-O	This work
$Li_{2.911}Er_{1.03}Cl_6$	1.7353	3.2344	3.0000	3.5928	hcp-O	This work

Supplementary Table 14. Typical two points based on the proposed cationic polarization factor of $Li_{3-3x}(Ho_{1-y}In_y)_{1+x}Cl_6$ halides along the line through *hcp-T* typed Li_3HoCl_6 to *ccp-M* typed $Li_{2.727}In_{1.091}Cl_6$.

Halides	$\tau = (\Phi Li + \Sigma\Phi M)/\Sigma\phi X$	$\phi Li = n_{Li}\cdot I_{Li}$ (Å^{-1})	$\phi M = n_M\cdot I_M$ (Å^{-1})	$\phi X = n_X\cdot I_X$ (Å^{-1})	Structure	Ref.
Li_3HoCl_6	1.7300	3.3333	2.8818	3.5928	hcp-T	[6]
$Li_{2.93}(Ho_{0.8}In_{0.2})_{1.023}Cl_6$	1.7448	3.2555	3.0131	3.5928	hcp-O	This work
$Li_{2.85}(Ho_{0.5}In_{0.5})_{1.05}Cl_6$	1.7688	3.1666	3.1884	3.5928	ccp-M	This work
$Li_{2.727}In_{1.091}Cl_6$	1.8125	3.3333	3.4819	3.5928	ccp-M	This work

Supplementary Table 15. Calculated molar content proportionally ionic potential of different ions and corresponding cationic polarization factors (before and after modification) for $Li_{3-x}M_{1-x}^{3+}M_x^{4+}X_6$ halides.

Halides	$\tau = (\Phi Li + \Sigma\Phi M)/\Sigma\phi X$	Modified $\tau = \frac{6-x}{6}(\Phi Li + \Sigma\Phi M)/\Sigma\phi X$	$\phi Li = n_{Li}\cdot I_{Li}$ ($Å^{-1}$)	$\phi M = n_M\cdot I_M$ ($Å^{-1}$)	$\phi X = n_X\cdot I_X$ ($Å^{-1}$)	Structure	Ref.
$Li_{2.8}Yb_{0.8}Zr_{0.2}Cl_6$	1.7876	1.728	3.1111	3.3112	3.5928	hcp-O	[22]
$Li_{2.75}Yb_{0.75}Hf_{0.25}Cl_6$ (500 °C)	1.7992	1.7146	3.0555	3.4086	3.5928	hcp-O	[21]
$Li_{2.7}Yb_{0.7}Zr_{0.3}Cl_6$	1.8033	1.7131	3	3.4787	3.5928	hcp-O	[22]
$Li_{2.633}Er_{0.633}Zr_{0.367}Cl_6$	1.8026	1.6923	2.9255	3.5507	3.5928	hcp-O	[23]
$Li_{2.5}Yb_{0.5}Zr_{0.5}Cl_6$	1.8346	1.6818	2.7778	3.8137	3.5928	hcp-O	[22]
$Li_{2.5}Y_{0.5}Zr_{0.5}Cl_6$	1.8219	1.6701	2.7778	3.7679	3.5928	hcp-O	[23]
$Li_{2.4}Er_{0.4}Zr_{0.6}Cl_6$	1.8432	1.6589	2.6666	3.9558	3.5928	hcp-O	[23]
$Li_{2.4}Y_{0.4}Zr_{0.6}Cl_6$	1.8401	1.6561	2.6666	3.9446	3.5928	hcp-O	[23]

Supplementary Table 16. Cation radius differences in $Li_3M^{3+}Cl_6$ chlorides.

	Halides	Structure	Cation radius difference between M^{3+} and Li^+ (Å)
Smallest cation radius difference	Li_3InCl_6	ccp-M	0.04
	Li_3ScCl_6	ccp-M	0.015
Moderate cation radius difference	Li_3YbCl_6	hcp-O	0.108
	Li_3LuCl_6	hcp-O	0.101
Largest cation radius difference	Li_3YCl_6	hcp-T	0.104
	Li_3TbCl_6	hcp-T	0.1063
	Li_3DyCl_6	hcp-T	0.1052
	Li_3HoCl_6	hcp-T	0.104
	Li_3ErCl_6	hcp-T	0.103
	Li_3TmCl_6	hcp-T	0.102

Supplementary Table 17. Cation radius differences in $Li_{3-x}M_{1-x}^{3+}M_x^{4+}X_6$ halides.

Halides		Cation radius difference between M^{3+} and Li^+(Å)	Cation radius difference between M^{4+} and Li^+(Å)	Cation radius difference between M^{3+} and M^{4+}(Å)
Small cation radius differences	$Li_{3-x}In_{1-x}Zr_xCl_6$	0.04	-0.04	0.08
	$Li_{3-x}Sc_{1-x}Zr_xCl_6$	-0.015	-0.04	0.0025
	$Li_{3-x}In_{1-x}Hf_xCl_6$	0.04	-0.05	0.09
Large cation radius differences	$Li_{2.8}Yb_{0.8}Zr_{0.2}Cl_6$	**0.108**	-0.04	**0.148**
	$Li_{2.75}Yb_{0.75}Hf_{0.25}Cl_6$ (500 °C)	**0.108**	-0.05	**0.158**
	$Li_{2.7}Yb_{0.7}Zr_{0.3}Cl_6$	**0.108**	-0.04	**0.148**
	$Li_{2.633}Er_{0.633}Zr_{0.367}Cl_6$	**0.13**	-0.04	**0.17**
	$Li_{2.5}Yb_{0.5}Zr_{0.5}Cl_6$	**0.108**	-0.04	**0.148**
	$Li_{2.5}Y_{0.5}Zr_{0.5}Cl_6$	**0.14**	-0.04	**0.18**
	$Li_{2.4}Er_{0.4}Zr_{0.6}Cl_6$	**0.13**	-0.04	**0.17**
	$Li_{2.4}Y_{0.4}Zr_{0.6}Cl_6$	**0.14**	-0.04	**0.18**

Supplementary Table 18. Calculated molar content proportionally ionic potential of different ions and corresponding cationic polarization factors (before and after modification) for $Li_{3-x}M_{1-x}^{3+}M_{x}^{4+}X_6$ halides.

Sample No.	Halides	Structure	$\tau = (\Phi Li + \Sigma\Phi M)/\Sigma\phi X$	Modified $\tau = \frac{6-x}{6}(\Phi Li + \Sigma\Phi M)/\Sigma\phi X$	$\phi Li = n_{Li}\cdot I_{Li}$ (Å^{-1})	$\phi M = n_M\cdot I_M$ (Å^{-1})	$\phi X = n_X\cdot I_X$ (Å^{-1})	Conductivity (S cm^{-1}, 25 °C)
1	$Li_{2.5}Yb_{0.5}Zr_{0.5}Cl_6$	hcp-O	1.8346	1.6818	2.7778	3.8137	3.5928	1.63×10^{-3}
2	$Li_{2.6}Y_{0.6}Hf_{0.4}Cl_6$	hcp-O	1.8097	1.6891	2.8889	3.6131	3.5928	1.31×10^{-3}
3	$Li_{2.7}Er_{0.7}Zr_{0.3}Cl_6$	hcp-O	1.7909	1.7104	3.0000	3.4342	3.5928	1.35×10^{-3}
4	$Li_{2.4}Ho_{0.4}Hf_{0.6}Cl_6$	hcp-O	1.8489	1.6640	2.6666	3.9763	3.5928	6.81×10^{-4}

Supplementary References

1 Tyagi, A. K., Köhler, J., Balog, P. & Weber, J. Syntheses and structures of Li_3ScF_6 and high pressure $LiScF_4$–, luminescence properties of $LiScF_4$, a new phase in the system LiF–ScF_3. *J. Solid State Chem.* **178**, 2620-2625, (2005).

2 Walker, P. Melt growth of rare-earth binary and complex halides. *Prog. Cryst. Growth Charact. Mater.* **3**, 103-119 (1980).

3 Xun, X., Feng, S. & Xu, R. Hydrothermal synthesis of complex fluorides $LiHoF_4$ and $LiErF_4$ with scheelite structures under mild conditions. *Mater. Res. Bull.* **33**, 369-375 (1998).

4 Li, X. *et al.* Water-Mediated Synthesis of a Superionic Halide Solid Electrolyte. *Angew. Chem. Int. Ed.* **58**, 16427-16432, (2019).

5 Liang, J. *et al.* Site-occupation-tuned superionic Li_xScCl_{3+x} halide solid electrolytes for all-solid-state batteries. *J. Am. Chem. Soc.***142**, 7012-7022 (2020).

6 Bohnsack, A. *et al.* Ternäre Halogenide vom Typ A_3MX_6. VI [1]. Ternäre Chloride der Selten-Erd-Elemente mit Lithium, Li_3MCl_6 (M=Tb-Lu, Y, Sc): Synthese, Kristallstrukturen und Ionenbewegung. *Z. Anorg. Allg. Chem.* **623**, 1067-1073 (1997).

7 Shannon, R. D. Revised effective ionic radii and systematic studies of interatomic distances in halides and chalcogenides. *Acta Crystallogr. A* **32**, 751-767 (1976).

8 Wang, K. *et al.* A cost-effective and humidity-tolerant chloride solid electrolyte for lithium batteries. *Nature Communications* **12**, 1-11 (2021).

9 Yamada, K., Kumano, K. & Okuda, T. Lithium superionic conductors Li_3InBr_6 and $LiInBr_4$ studied by ^{7}Li, ^{115}In NMR. *Solid State Ionics* **177**, 1691-1695 (2006).

10 Bhoyar, R. A., Nayak, A., Tawalare, P., Wankhede, S. & Moharil, S. in *Journal of Physics: Conference Series.* 012036 (IOP Publishing).

11 Bohnsack, A., Balzer, G., Güdel, H. U., Wickleder, M. S. & Meyer, G. Ternäre Halogenide vom Typ A_3MX_6. VII [1]. Die Bromide Li_3MBr_6 (M= Sm-Lu, Y): Synthese, Kristallstruktur, Ionenbeweglichkeit. *Z. Anorg. Allg. Chem.* **623**, 1352-1356 (1997).

12 Shi, X. *et al.* Fast Li-ion Conductor of Li_3HoBr_6 for Stable All-Solid-State Lithium–Sulfur Battery. *Nano Lett.* **21**, 9325-9331, (2021).

13 Lachgar, A., Dudis, D. S., Dorhout, P. K. & Corbett, J. D. Synthesis and properties of two novel line phases that contain linear scandium chains, lithium scandium iodide ($LiScI_3$) and sodium scandium iodide ($Na_{0.5}ScI_3$). *Inorg. Chem.* **30**, 3321-3326 (1991).

14 Roman, S. *et al.* A Lattice Dynamical Approach for Finding the Lithium Superionic Conductor Li_3ErI_6. *ACS Appl. Energy Mater.* **3**, 3684-3691 (2020).

15 Mukhametshina, Z., Parakhin, V., Seleznev, V. & Chekmarev, A. X-ray diffraction characteristics of hexaiodozirconate and hexaiodohafnate of lithium. *Zh. Neorg. Khim.* **31**, 2187-2189 (1986).

16 Li, X. *et al.* Origin of Superionic $Li_3Y_{1-x}In_xCl_6$ Halide Solid-Electrolytes with High Humidity Tolerance. *Nano Lett.* **20**, 4384-4392 (2020).

17 Liu, Z. *et al.* High Ionic Conductivity Achieved in $Li_3Y(Br_3Cl_3)$ Mixed Halide Solid Electrolyte via Promoted Diffusion Pathways and Enhanced Grain Boundary. *ACS Energy Lett.* **6**, 298-304 (2020).

18 Asano, T. *et al.* Solid halide electrolytes with high lithium-ion conductivity for application in 4 V class bulk-type all-solid-state batteries. *Adv. Mater.* **30**, 1803075 (2018).

19 Liang, J. *et al.* A Series of Ternary Metal Chloride Superionic Conductors for High-Performance All-Solid-State Lithium Batteries. *Adv. Energy Mater.* **12**, 2103921, (2022).
20 Schlem, R. *et al.* Mechanochemical Synthesis: A Tool to Tune Cation Site Disorder and Ionic Transport Properties of Li_3MCl_6 (M = Y, Er) Superionic Conductors. *Adv. Energy Mater.* **10**, 1903719 (2020).
21 Park, J. *et al.* Heat Treatment Protocol for Modulating Ionic Conductivity via Structural Evolution of $Li_{3-x}Yb_{1-x}M_xCl_6$ (M= Hf^{4+}, Zr^{4+}) New Halide Superionic Conductors for All-Solid-State Batteries. *Chem. Eng. J.* **425**, 130630 (2021).
22 Kim, S. Y. *et al.* Lithium ytterbium-based halide solid electrolytes for high voltage all-solid-state batteries. *ACS Mater. Lett.* **3**, 930-938 (2021).
23 Park, K.-H. *et al.* High-voltage superionic halide solid electrolytes for all-solid-state Li-ion batteries. *ACS Energy Lett.* **5**, 533-539 (2020).
24 Yu, T. *et al.* Superionic Fluorinated Halide Solid Electrolytes for Highly Stable Li-Metal in All-Solid-State Li Batteries. *Adv. Energy Mater.* **11**, 2101915 (2021).
25 Kwak, H. *et al.* New Cost-Effective Halide Solid Electrolytes for All-Solid-State Batteries: Mechanochemically Prepared Fe^{3+}-Substituted Li_2ZrCl_6. *Adv. Energy Mater.* **11**, 2003190 (2021).
26 Luo, X. *et al.* Heterovalent Cation Substitution to Enhance the Ionic Conductivity of Halide Electrolytes. *ACS Applied Materials & Interfaces* **13**, 47610-47618 (2021).
27 Helm, B. *et al.* Exploring Aliovalent Substitutions in the Lithium Halide Superionic Conductor $Li_{3-x}In_{1-x}Zr_xCl_6$ ($0 \leq x \leq 0.5$). *Chem. Mater.* **33**, 4773-4782 (2021).
28 Kwak, H. *et al.* Li^+ conduction in aliovalent-substituted monoclinic Li_2ZrCl_6 for all-solid-state batteries: $Li_{2+x}Zr_{1-x}M_xCl_6$ (M= In, Sc). *Chem. Eng. J.* **437**, 135413 (2022).
29 Tomita, Y., Ohki, H., Yamada, K. & Okuda, T. Ionic conductivity and structure of halocomplex salts of group 13 elements. *Solid State Ionics* **136**, 351-355 (2000).
30 Plass, M. A., Bette, S., Dinnebier, R. E. & Lotsch, B. V. Enhancement of Superionic Conductivity by Halide Substitution in Strongly Stacking Faulted $Li_3HoBr_{6-x}I_x$ Phases. *Chem. Mater.* **34**, 3227-3235 (2022).
